# Repurposing Tyrosine Kinase Inhibitors to Overcome Multidrug Resistance in Cancer: A Focus on Transporters and Lysosomal Sequestration

**DOI:** 10.3390/ijms21093157

**Published:** 2020-04-30

**Authors:** Maria Krchniakova, Jan Skoda, Jakub Neradil, Petr Chlapek, Renata Veselska

**Affiliations:** 1 Laboratory of Tumor Biology, Department of Experimental Biology, Faculty of Science, Masaryk University, 61137 Brno, Czech Republic; maria.krchniakova@mail.muni.cz (M.K.); jan.skoda@sci.muni.cz (J.S.); jneradil@sci.muni.cz (J.N.); chlapek@sci.muni.cz (P.C.); 2 International Clinical Research Center, St. Anne’s University Hospital, 65691 Brno, Czech Republic

**Keywords:** tyrosine kinase inhibitor, multidrug resistance, cancer, ABC transporter, SLC transporter, lysosomal sequestration

## Abstract

Tyrosine kinase inhibitors (TKIs) are being increasingly used to treat various malignancies. Although they were designed to target aberrant tyrosine kinases, they are also intimately linked with the mechanisms of multidrug resistance (MDR) in cancer cells. MDR-related solute carrier (SLC) and ATB-binding cassette (ABC) transporters are responsible for TKI uptake and efflux, respectively. However, the role of TKIs appears to be dual because they can act as substrates and/or inhibitors of these transporters. In addition, several TKIs have been identified to be sequestered into lysosomes either due to their physiochemical properties or via ABC transporters expressed on the lysosomal membrane. Since the development of MDR represents a great concern in anticancer treatment, it is important to elucidate the interactions of TKIs with MDR-related transporters as well as to improve the properties that would prevent TKIs from diffusing into lysosomes. These findings not only help to avoid MDR, but also help to define the possible impact of combining TKIs with other anticancer drugs, leading to more efficient therapy and fewer adverse effects in patients.

## 1. Introduction

Tyrosine kinase inhibitors (TKIs) are low molecular weight (<800 Da) organic compounds that are able to penetrate the cell membrane and interact with targets inside the cell. They were developed to block the ATP-binding sites of protein tyrosine kinases, thereby inhibiting or attenuating the enzymatic activity of aberrant tyrosine kinases responsible for the malignant phenotype of cells. Such targeted therapy can be aimed at either cancer cells, by inhibiting their proliferation and affecting their susceptibility to apoptosis, or the tumor microenvironment, by affecting angiogenesis and the invasion or formation of metastases.

So far, a number of TKIs has been approved by FDA for clinical use (respective molecular targets are summarized in Supplementary Table S1), but many more are currently under investigation: a brief example of experimental TKIs is listed in Supplementary Table S2. Due to their convenient oral administration, TKIs are used not only in anticancer therapy, but also in treating diabetes, inflammation, severe bone disorders and arteriosclerosis [1,2,3]. 

However, even anticancer treatment using TKIs leads to the development of multidrug resistance (MDR), i.e., resistance to structurally and functionally different drugs [4]. Thus, the main focus of this review is to describe the noncanonical role of TKIs in selected MDR mechanisms, which involve membrane transporters and drug accumulation in lysosomes.

## 2. Effects of TKIs on Membrane Transporters

The ATP-binding cassette (ABC) and the solute carrier (SLC) membrane transporters are considered to be the most relevant transporters affecting the exposure to administered TKIs [4]. Both types are expressed ubiquitously throughout human tissue and can recognize and translocate various molecules across biological membranes, including TKIs. As such, they can affect the pharmacokinetic parameters of TKIs, such as drug absorption, distribution, metabolism, excretion and toxicity [4]. Therefore, these transporters expressed on cancer cells are considered a major determinant of MDR because increased efflux or decreased transporter-mediated influx can lead to inefficient intracellular drug concentrations and/or undesired drug interactions.

### 2.1. ABC Transporters

ABC transporters are transmembrane proteins that have been investigated in relation to their active drug efflux irrespective of the prevailing gradient, thus causing drug resistance. The most widely studied ABC transporters with respect to MDR include P-glycoprotein (Pgp, ABCB1), multidrug resistance protein 1 (MRP1, ABCC1) and breast cancer resistance protein (BCRP, ABCG2). Multiple mechanisms modulating the expression of ABC transporters have been proposed [5], including the loss of uL3 ribosomal protein, which has been recently associated with the upregulation of ABCB1 [6]. An overview of ABC transporters involved in MDR and interacting with TKIs is listed in Table 1.

Some TKIs are able to bind to the substrate-binding pocket of an ABC transporter (Table 1) [7,8,9,10,11], which leads to their efflux from cells and explains the reduced therapeutic efficacy and/or resistance acquired during the course of TKI therapy. ABCA3 protected leukemic stem cells from dasatinib, imatinib, and nilotinib, which target the BCR-ABL kinase [7]. Exposure to these TKIs led to a dose-dependent increase in ABCA3 transcription, supporting drug efflux, but when cells were cotreated with the COX2 inhibitor indomethacin, ABCA3 expression decreased, and the combination potentiated the antineoplastic efficacy of TKIs [7]. Similarly, gefitinib causes indirect induction of ABCG2 expression [12]. In fact, targeting EGFR with gefitinib results in its internalization, phosphorylation by Akt and translocation to the nucleus, where EGFR affects the *ABCG2* gene promoter enhancing its transcription [12].

In contrast, TKIs can also act as inhibitors of ABC transporters. Similarly to their interaction with protein tyrosine kinases, TKIs block the ATP-binding sites of membrane transporters, preventing the phosphorylation and inhibiting the efflux function of transporters [13,14,15,16,17]. Although cabozantinib affected the ATPase activity of the ABCG2 transporter, it also interacted with the transporter at the drug-substrate binding site, antagonizing the transporter by competitive inhibition [15]. TKIs usually inhibit ABC transporters directly and do not alter their expression or localization [13,16,17]. 

Interestingly, ponatinib treatment resulted in a decrease in ABCB1 and ABCG2 cell surface expression, and imatinib downregulated ABCG2 expression in BCR-ABL-positive cells [18,19]. However, these effects were most likely caused indirectly via inhibition of the Akt signaling that is downstream of the BCR-ABL axis that is inhibited by the TKIs [18,19].

When inhibiting ABC transporters, substrate drugs are no longer pumped outside of cells, and the cytotoxicity of substrate drugs in resistant cells overexpressing ABC transporters is significantly increased. In vitro studies demonstrated that TKI administration increased intracellular accumulation of rhodamine 123 or doxorubicin in multidrug-resistant cells overexpressing selected ABC transporters [20,21]. Treatment with TKIs inhibiting these transporters (Table 1) was able to enhance the cytotoxicity of substrate drugs, such as paclitaxel, docetaxel [14], vincristine, vinblastine [20,22], doxorubicin [20], etoposide [23], cytarabine [24], mitoxantrone and topotecan [15,19,25], while sensitivity to cisplatin, which is not a substrate for ABC transporters, was not significantly altered [26]. The inhibitory effect of TKIs (e.g., gefitinib or ibrutinib) was comparable to that of known inhibitors of ABC transporters [14,27]. Resensitizing multidrug-resistant cancer cells can also be achieved by combining a TKI with an ABC transporter substrate affinity together with a second TKI having an ABC transporter inhibitory activity. A low-dose treatment with the ABCB1 transporter substrate dasatinib, in combination with the ABCB1 inhibitor nilotinib, provided additive/synergistic effects in leukemic cells overexpressing ABCB1 [28]. Supporting these findings, in in vivo experiments in respective xenograft mouse models, TKIs combined with conventional chemotherapeutics showed a greater inhibitory effect on tumor growth than single drugs [20,29,30]. Furthermore, simultaneous inhibition of ABCB1 and ABCG2 by erlotinib at the mouse blood–brain barrier improved brain permeability and pazopanib accumulation [31].

**Figure 1:**
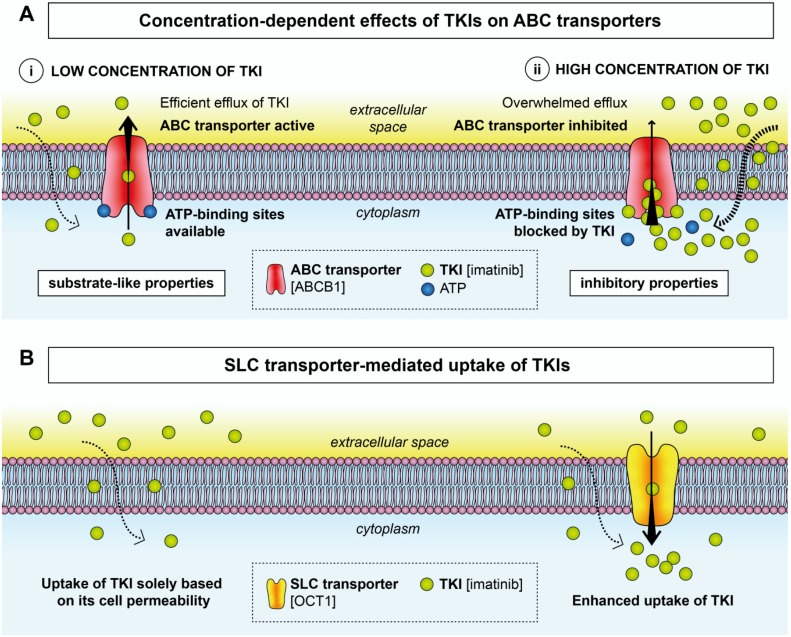
Transport of TKIs by ABC and SLC transporters. (**A**) At low concentrations (**i**), some TKIs exhibit substrate-like properties and are exported out of the cell by the respective ABC transporters. A high concentration of TKIs (**ii**) leads to blockage of the ATP-binding sites of ABC transporters, which results in inhibited efflux of the TKI. (**B**) Upregulated expression of SLC transporters can lead to enhanced uptake of some TKIs. Examples of TKIs and specific transporters are given in square brackets.

Depending on their concentration and affinity for the transporter, a number of TKIs have been reported to interact with ABC transporters as both substrates and inhibitors (Figure 1A) [17,19,25,32,33]. At lower concentrations, TKIs usually possess substrate-like properties (Figure 1Ai), but they tend to act as ABC inhibitors at higher yet pharmacologically relevant concentrations (Figure 1Aii) [13,19]. Indeed, combining ponatinib with topotecan or mitoxantrone, substrates of both ABCB1 and ABCG2, resulted in antagonistic effects at lower ponatinib concentrations, whereas higher concentrations led to synergistic effects [19]. In addition, contradictory effects have also been attributed to pazopanib. While it was described as a substrate for both ABCB1 and ABCG2 in the canine kidney cell line MDCKII [31], another study reported that pazopanib was an ABCB1 inhibitor that inhibited dasatinib efflux from LLC-PK1 porcine kidney cells [34].

### 2.2. SLC Transporters

While ABC transporters harness energy from ATP hydrolysis and function as efflux transporters, SLC transporters are primarily involved in the uptake of small molecules into cells, including TKIs [62] (Figure 1B). Unlike the described MDR mediated by ABC transporters in a number of malignancies, knowledge about the interactions of SLC transporters with drugs used in anticancer treatment is limited. Table 2 contains an overview of TKIs known to interact with SLC transporters.

The activity of imatinib was linked with the expression of organic cation transporter 1 (OCT1, SLC22A1), as it was found to be a substrate for this transporter in the CEM human leukemia cell line [73]. A positive correlation was found in patients with chronic myeloid leukemia (CML) in a phase II trial between survival and the functional activity of OCT1 that was assessed by measuring imatinib influx [63,64]. In addition, temperature-dependent uptake experiments demonstrated that the uptake of imatinib was an active process rather than a passive penetration of cell membranes [73]. Other transporters that might affect the oral absorption of imatinib and the liver access of imatinib include the uptake organic cation/carnitine transporter (OCTN2, SLC22A5) and the uptake organic anion-transporting polypeptides OATP1A2 (SLCO1A2) and OATP1B3 (SLCO1B3), for which imatinib is a substrate [8].

In contrast, the cellular uptake of nilotinib seems to be independent of OCT expression. This was observed in KCL22 human leukemia cell line overexpressing OCT1 [66] as well as in mononuclear cells from patients with CML [74]. In fact, nilotinib has been reported as a potential inhibitor of OCT1 [66], OCT2, OCT3 [65] and OATP1B1 [71].

The uptake of drugs into nontarget (nonneoplastic) cells by SLC transporters resulting in higher drug toxicity presents another obstacle in anticancer treatment. Organic anion transporter 6 (OAT6, SLC22A20) was found to regulate the entry of sorafenib into keratinocytes, contributing to sorafenib-induced skin toxicity [70].

## 3. Lysosomal Sequestration

**Figure 2:**
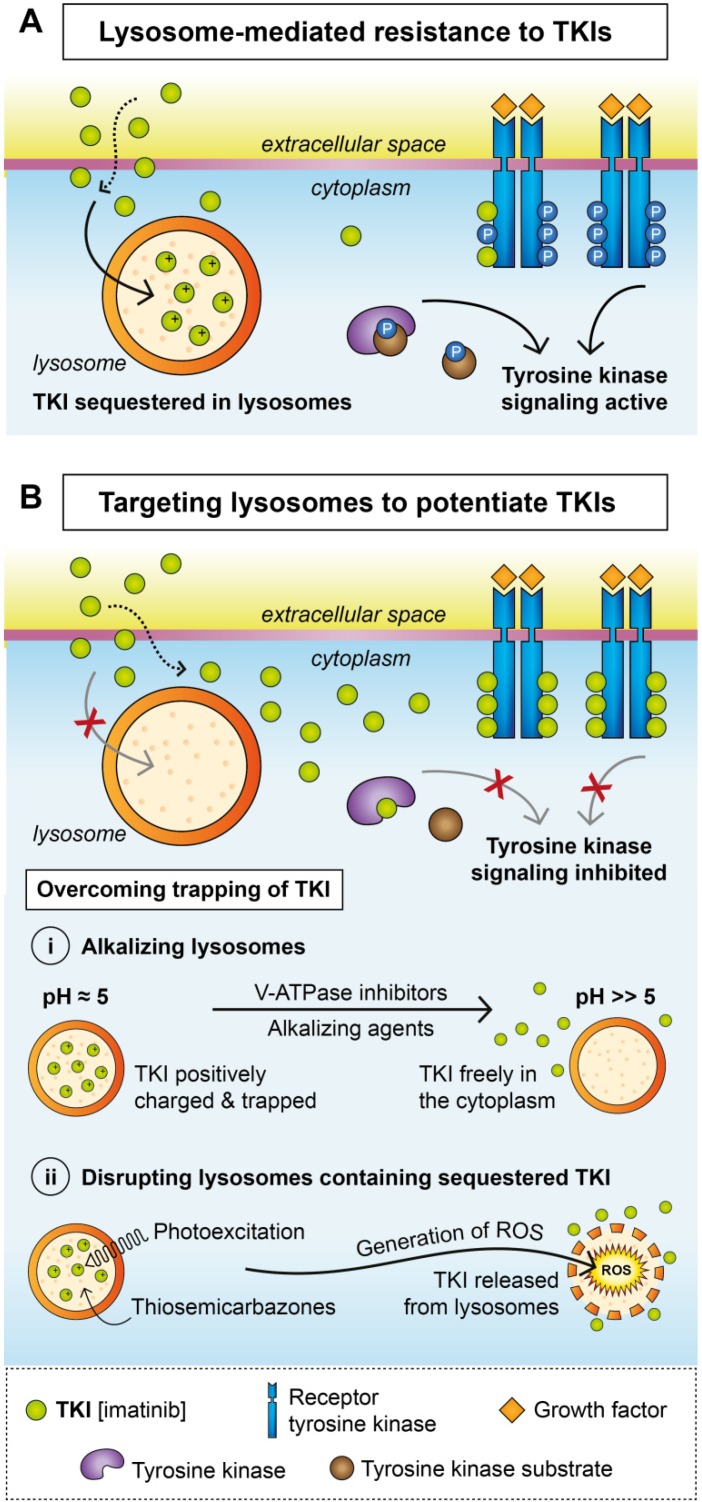
Lysosomes in resistance to TKIs. (**A**) Sequestration of TKIs into lysosomes provides a mechanism of resistance to TKIs. (**B**) Targeting lysosomes by alkalizing their milieu (**i**) or disrupting their integrity (**ii**) can potentiate the effects of TKI treatment.

Lysosomes contribute to MDR via a mechanism called lysosomal trapping. Compounds can be sequestered (trapped) in lysosomes based on their physiochemical properties: (i) basic pKa, an acid dissociation constant for the conjugated acid of the weak base, which affects the extent of lysosomal trapping, and (ii) logP, the partition coefficient between octanol and water, which regulates the kinetics of passive membrane permeability [75]. Accumulation in lysosomes is typical for lipophilic and amphiphilic compounds with lipophilic amines (logP > 1) and weak bases with ionizable amine groups (pKa > 6) [75]. Due to their hydrophobic character, these drugs are able to permeate the lipid membranes via passive diffusion. However, after entering the acidic environment of lysosomes, compounds become positively charged, which restricts their diffusion back into the cytoplasm and prevents them from reaching their cytoplasmic or nuclear targets [75] (Figure 2A). Furthermore, lysosomal sequestration is driven by the pH difference between the neutral cytosol (pH ~ 7.2) and the acidic lysosomal compartment (pH ~ 5) [76]. This process requires continuous acidification of the lysosomes by membrane-bound ATP-dependent lysosomal proton pumps of the vacuolar ATPase (V-ATPase) family. Agents that are sequestered in lysosomes are called lysosomotropic, and their accumulation within lysosomes is known as lysosomotropism [75].

Lysosomal sequestration has been recognized as another mechanism of resistance to TKIs [77], and TKIs known to be accumulated in lysosomes are summarized in Table 3. The ability of TKIs to be sequestered in lysosomes can be detected by fluorescence microscopy in the case of inhibitors that exhibit autofluorescence, such as sunitinib [23,77], lapatinib [78], imatinib [79,80] or nintedanib [81], and they colocalize with stained lysosomes. In the case of TKIs that are not autofluorescent (e.g., gefitinib or lapatinib), lysosomal sequestration can be demonstrated by their influence on the lysosomal accumulation of LysoTracker^®^ Red [76].

Several TKIs do not harbor physiochemical properties of hydrophobic, weak base molecules but can be entrapped in the acidic milieu of lysosomes (Table 3) [82,83,84]. ABC transporters facilitate the active accumulation of drugs in lysosomes, as these pumps have been found on the membranes of intracellular compartments, including the Golgi apparatus and intracellular vesicles [85,86]. ABCA3 [87], ABCB1 [88], and ABCG2 [89] were demonstrated on lysosomal membranes, explaining the lysosomal sequestration of their respective substrate TKIs, including imatinib [87], sorafenib [83] and pazopanib [84].

Interestingly, the ABCB1-mediated resistance phenotype of leukemia cells was stronger when ABCB1 was expressed intracellularly than when it was expressed on the plasma membrane, indicating that the accumulation of drugs in lysosomes is most likely more effective than the efflux via membrane transporters [85]. Furthermore, stressors present in the tumor microenvironment (e.g., hypoxia, oxidants, or glucose starvation) were found to upregulate and relocalize ABCB1 to lysosomal membranes, resulting in increased drug resistance [88].

In many cases, resistance mediated by lysosomal sequestration is reversible. Removing sunitinib from tumor cell culture for several weeks resulted in normalization of cell lysosomal capacity and recovery of drug sensitivity [77]. Similarly to the in vitro data, patients with metastatic renal cell carcinoma developed resistance to sunitinib. However, it was transient after treatment interruption and subsequent rechallenge [90].

### Overcoming Lysosomal Sequestration

There are several mechanisms that may reverse sequestration: either preventing the accumulation of TKIs in the lysosomes by alkalizing the lysosomal milieu or disrupting the lysosomal membrane leading to efflux of TKIs. Concomitant or sequential treatment with TKIs and drugs that interfere with lysosomal function could present an effective means of overcoming the MDR mediated by lysosomal trapping (Figure 2B).

Several alkalizing agents have been introduced to circumvent lysosomal trapping (Figure 2Bi). Bafilomycin A1 targets V-ATPase, an enzyme that acidifies lysosomes during biogenesis, and was reported to sensitize cells towards previously sequestered nintedanib [81]. Although it prevents lysosomal sequestration in vitro, efficient concentrations of bafilomycin A1 also exert cytotoxicity in normal cells, which hinders its use in clinical settings [77]. Chloroquine, originally established as an antimalarial agent, accumulates in lysosomes, increases lysosomal pH and triggers destabilization of the lysosomal membrane. Combined treatment using chloroquine and sunitinib resulted in enhanced inhibition of tumor growth in a xenograft mouse model [91]. Similarly, the chloroquine analogues hydroxychloroquine and Lys05 have been shown to target lysosome-mediated autophagy and have been tested with other anticancer therapies [92,93,94].

Interestingly, sunitinib itself is able to reduce the activity of acid sphingomyelinase that promotes lysosomal membrane stability, leading to destabilization of lysosomes and inducing nonapoptotic lysosome-dependent cell death [23].

Photodestruction of lysosomes with sequestered photoexcitable TKIs presents another approach for overcoming lysosomal trapping (Figure 2Bii). Exposing sequestered sunitinib to a specific wavelength in vitro resulted in the generation of reactive oxygen species (ROS) and almost immediate disruption of lysosomes, followed by the release of the drug into the cytoplasm [95]. Similar observations and markedly attenuated tumor growth were reported after sunitinib photoexcitation in a xenograft model [95].

However, phototherapy is limited due to superficial and local treatment options, and apart from chloroquine [91], not many effective drugs have been identified to accumulate in lysosomes and then disrupt the lysosomal membrane. Thiosemicarbazone iron chelators represent novel anticancer agents that are transported into the lysosomes via ABCB1 [96]. There, they create redox-active complexes with copper, and generated ROS permeate the lysosomal membrane (Figure 2Bii). Thiosemicarbazones were able to disrupt lysosomes and free sequestered doxorubicin in ABCB1-overexpressing cells [88,96]. Whether these agents can potentiate the effect of TKIs trapped in lysosomes is yet to be elucidated.

## 4. Clinical Trials Repurposing TKIs in Combinational Strategies

The ability of several TKIs to modulate ABC transporters was shown in cancer cell lines as well as in xenograft models and primary cells collected from patients [29,33,40,49]. TKIs inhibiting ABC transporters were able to reverse the MDR phenotype of cancer cells and enhance the effect of other anticancer drugs at the quite low, usually noncytotoxic concentrations achieved in patients [13,20,56]. This evidence underlines the potential clinical value of TKIs and provides a rationale for their repurposing in combinational strategies overcoming ABC transporter-mediated MDR. Table 4 lists examples of clinical trials combining TKIs with other anticancer drugs.

Promising efficacy and improved clinical outcomes were described when combining paclitaxel with neratinib [112] or lapatinib [110] in HER2-positive breast cancer patients. Favorable results were also observed in combinations of docetaxel with nintedanib in non-small-cell lung carcinoma patients [114] and with sunitinib in patients with gastric cancer [120]. Resistance to docetaxel and paclitaxel is often caused by ABCB1- and ABCC10-mediated efflux [26]. Hence, adding TKIs that inhibit these transporters (Table 1), e.g., applied lapatinib [16,48], neratinib [29], nintedanib [22], or sunitinib [21], could, in fact, decrease the efflux of chemotherapeutics and result in enhanced antitumor effects observed in the studies (Table 4).

Similar conclusions could be drawn from the trials that combined erlotinib and gemcitabine for the treatment of advanced pancreatic cancer [105,106]. Gemcitabine plus erlotinib showed additive efficacy compared to gemcitabine alone [105] and addition of oxaliplatin to this regimen resulted in higher response rate and improved progression-free survival [106]. In vitro studies revealed that the resistance to oxaliplatin develops after upregulation of ABCC1 and ABCC4 transporters [123]. Furthermore, a combined siRNA-mediated knockdown of *ABCC3*, *ABCC5*, and *ABCC10* significantly sensitized cells to gemcitabine [124]. As erlotinib was demonstrated as a potent inhibitor of multiple ABC transporters (Table 1), including those that efflux gemcitabine and oxaliplatin from cancer cells [16,54] and are known to cause resistance in pancreatic adenocarcinomas [125], these data possibly elucidate the increased efficacy of the combined treatment in the respective clinical trials [105,106].

These examples demonstrate that TKIs added to the treatment enhance the response by not only targeting aberrant tyrosine kinases in malignant cells but also by sensitizing resistant tumors to other anticancer agents. Although the clinical trials (Table 4) were focused on advanced, metastatic and/or recurrent malignancies with known resistance to therapy, not all drug combinations attained satisfactory outcomes in patients [103,107,115,116,118]. However, the mechanisms mediating MDR in tumors were usually not examined and most trials did not focus specifically on reversing the ABC transporter-mediated MDR. This urges the need for combination strategies that would select TKIs attentively with regard to multiple determinants, including the tumor type, its expression profile as well as presence of MDR-related factors, in order to tailor therapeutic regiments that may lead to overcoming resistance and improved clinical response.

Nanotechnology could present a valuable strategy in combining TKIs with conventional chemotherapeutics [126]. Polymeric nanoparticles allowed a co-delivery of erlotinib and doxorubicin on the same platform while facilitating a sequential release of the drugs, which resulted in the enhanced cytotoxic effect on breast cancer cells [127]. Therefore, nanomedicine offers a convenient multidrug delivery system where the first released drug (TKI, e.g., erlotinib) sensitizes the cancer cells to the second drug (conventional chemotherapeutic, e.g., doxorubicin), hence avoiding MDR development and making therapy more efficient [126,127].

## 5. Conclusions

A more personalized approach to therapy, such as targeted therapy using TKIs, has been increasingly used in treating various types of malignancies. Emerging evidence suggests that apart from identifying specific targets of TKIs, it is also important to evaluate other characteristics of tumor cells as well as the drug itself. The expression of uptake/efflux membrane transporters and the physiochemical qualities of TKIs affect the exposure of administered TKIs.

**Figure 3:**
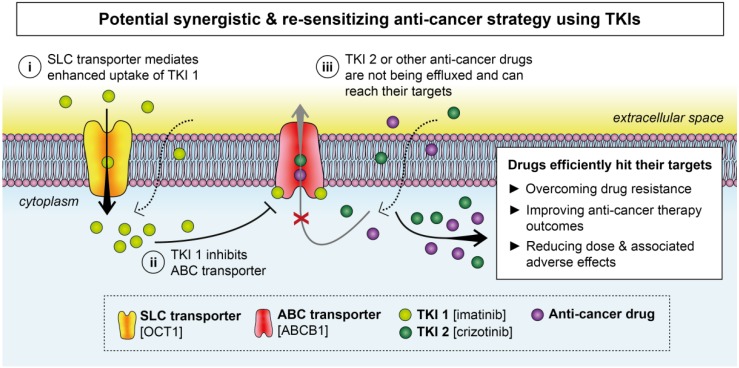
Schematic illustration of a potential anticancer strategy using TKIs that exploits the upregulated expression of SLC transporters to resensitize cells to anticancer drugs. In this scenario, high levels of certain SLC transporters (e.g., OCT1) are utilized to load a cancer cell with the first TKI (TKI 1; e.g., imatinib) (**i**). Apart from hitting its targets, TKI 1 also inhibits ABC transporters (e.g., ABCB1) (**ii**) so the second TKI (TKI 2; e.g., crizotinib) or other anti-cancer drugs are no longer effluxed from cancer cells (**iii**), which eventually results in synergistic effects of the drugs and improved treatment response.

Furthermore, the dual effects of TKIs on membrane transporters allow them to not only exert anticancer effects but also act as chemosensitizers to reverse the transporter-mediated efflux of other anticancer drugs. For instance, high expression of specific membrane transporters could provide the perfect environment for the therapeutic application of the TKIs that are transported into the cancer cells by abundant SLC transporters but at the same time inhibit the drug efflux pumps. This allows for either sequential or simultaneous administration of TKIs with other cytotoxic agents, harboring great synergistic potential, improving the efficacy of therapy, avoiding or reversing drug resistance, and possibly reducing associated toxicity and adverse effects (Figure 3).

## Figures and Tables

**Table 1:** Interactions of selected TKIs with ABC transporters.

ABC Transporter	Substrate	Inhibitor	Substrate/Inhibitor
**ABCA3**	dasatinib [7]; imatinib [7]; nilotinib [7]	–	–
**ABCB1** (P-glycoprotein, MDR1)	brigatinib [9]; crizotinib [35]	cabozantinib [36]; canertinib * [31]; cediranib * [37]; ceritinib [38]; erlotinib [34]; gefitinib [14]; motesanib * [39]; neratinib [29]; osimertinib [40]; regorafenib [34]; saracatinib * [41]; sorafenib [34]; sunitinib [21]; vandetanib [42]; vatalanib * [43]	afatinib [44]; alectinib [33]; apatinib * [17]; bosutinib [45]; dasatinib [45]; ibrutinib [27]; imatinib [46]; lapatinib [47,48]; nilotinib [45]; nintedanib [22]; pazopanib [31,34]; ponatinib [19]
**ABCC1** (MRP1)	–	cediranib * [37]; ibrutinib [49]; sunitinib [21]; vandetanib [42]	–
**ABCC2** (MRP2)	sorafenib [50]	sunitinib [51]	–
**ABCC3** (MRP3)	imatinib [52]; sorafenib [53]	–	–
**ABCC4** (MRP4)	imatinib [8]	erlotinib [54]; gefitinib [54]; sorafenib [55]; sunitinib [51]	–
**ABCC6** (MRP6)	dasatinib [10]; nilotinib [10]	–	–
**ABCC10** (MRP7)	gefitinib [11]	erlotinib [16]; ibrutinib [27]; imatinib [56]; lapatinib [16]; linsitinib * [13]; masitinib * [30]; nilotinib [20]; ponatinib [57]; sorafenib [24]	–
**ABCC11** (MRP8)	–	sorafenib [24]	–
**ABCG2** (BRCP)	brigatinib [9]; gefitinib [58]	axitinib [34]; cabozantinib [15]; canertinib * [31]; ceritinib [38]; erlotinib [34]; icotinib * [59]; linsitinib * [13]; masitinib * [60]; osimertinib [40]; quizartinib * [61]; regorafenib [34]; sorafenib [24]; sunitinib [21]; tandutinib * [15]; vandetanib [42]; vatalanib * [43]	afatinib [32]; alectinib [33]; apatinib * [17]; bosutinib [45]; dasatinib [45]; imatinib [46]; lapatinib [47]; nilotinib [45]; pazopanib [31,34]; ponatinib [19]; telatinib * [25]

* experimental TKIs.

**Table 2:** Interactions of selected TKIs with SLC transporters.

SLC Transporter	Substrate	Inhibitor
**OCT1** (SLC22A1)	imatinib [63,64] sorafenib [55]	crizotinib [51] erlotinib [65] gefitinib [65] nilotinib [66] sunitinib [65]
**OCT2** (SLC22A2)	erlotinib [67]	crizotinib [68] gefitinib [65] nilotinib [65] saracatinib [69] sunitinib [65] vandetanib [68]
**OCT3** (SLC22A3)	–	gefitinib [65] nilotinib [65] sunitinib [65]
**OCTN2** (SLC22A5)	imatinib [8]	–
**OAT3** (SLC22A8)	erlotinib [67]	–
**OAT6** (SLC22A20)	sorafenib [70]	–
**OATP1A2** (SLCO1A2)	imatinib [8]	–
**OATP1B1** (SLCO1B1)	–	axitinib [71] lapatinib [51] nilotinib [71] pazopanib [71] sorafenib [71]
**OATP1B3** (SLCO1B3)	imatinib [8]	–
**OATP2B1** (SLCO2B1)	erlotinib [72]	–

**Table 3:** List of TKIs known to be sequestered into lysosomes.

TKI	pKa ^1^	LogP ^2^	Reference
dasatinib	8.49	3.82	[82]
gefitinib	6.85	3.75	[76]
imatinib	8.10	4.50	[79]
lapatinib	7.20	4.64	[76]
nilotinib	6.30	5.36	[80]
nintedanib	7.90	3.60	[81]
pazopanib	5.07	3.60	[84]
sorafenib	4.34	2.03	[83]
sunitinib	9.04	5.20	[77]

^1^ acid dissociation constant for the conjugated acid of the weak base. ^2^ partition coefficient between octanol and water.

**Table 4:** Combinational strategies using TKIs in clinical trials.

Combination of Drugs		Malignancy	Reference
apatinib *	+ etoposide + irinotecan	ovarian cancer high-grade glioma	[97] [98]
cediranib *	+ carboplatin, paclitaxel + cisplatin, gemcitabine	cervical cancer biliary tract cancer	[99] [100]
crizotinib	+ methotrexate	NSCLC	[101]
erlotinib	+ cabozantinib + carboplatin + everolimus + gemcitabine + gemcitabine, oxaliplatin + topotecan	NSCLC ovarian carcinoma HNSCC pancreatic cancer pancreatic cancer solid tumors	[102] [103] [104] [105] [106] [107]
gefitinib	+ carboplatin, pemetrexed	NSCLC	[108]
lapatinib	+ capecitabine + paclitaxel	breast cancer breast cancer	[109] [110]
neratinib	+ capecitabine + paclitaxel	breast cancer breast cancer	[111] [112]
nilotinib	+ vincristine, daunorubucin	ALL	[113]
nintedanib	+ docetaxel	NSCLC	[114]
sorafenib	+ cytarabine, daunorubicin + doxorubicin + gemcitabine, cisplatin	AML hepatocellular carcinoma collecting duct carcinoma	[115] [116] [117]
sunitinib	+ capecitabine + docetaxel	breast cancer breast cancer, gastric cancer	[118] [119,120]
vandetanib	+ docetaxel + pemetrexed	urothelial cancer NSCLC	[121] [122]

* experimental TKIs; AML: acute myeloid leukemia; ALL: acute lymphoblastic leukemia; HNSCC: head and neck squamous cell carcinoma; NSCLC: non-small-cell lung carcinoma.

## Supplementary Materials

The following are available online at https://www.mdpi.com/1422-0067/21/9/3157/s1, Table S1: FDA-approved TKIs and their molecular targets, Table S2: An example of TKIs under investigation and their molecular targets.

## Abbreviations

ABC	ATP-binding cassette
ALL	acute lymphoblastic leukemia
AML	acute myeloid leukemia
BRCP	breast cancer resistance protein
CML	chronic myeloid leukemia
EGFR	epidermal growth factor receptor
HER2	human epidermal growth factor receptor 2
HNSCC	head and neck squamous cell carcinoma
LAMP2	lysosome-associated membrane protein 2
MDR	multidrug resistance
MRP	multidrug resistance protein
NSCLC	non-small-cell lung carcinoma
OAT	organic anion transporter
OATP	organic anion-transporting polypeptide
OCT	organic cation transporter
OCTN	organic cation/carnitine transporter
Pgp	P-glycoprotein
ROS	reactive oxygen species
SLC	solute carrier
TKI	tyrosine kinase inhibitor
V-ATPase	vacuolar ATPase
