# Supplementary material for: Repurposing Tyrosine Kinase Inhibitors to Overcome Multidrug Resistance in Cancer: A Focus on Transporters and Lysosomal Sequestration
Source: Int J Mol Sci. 2020 Apr 30;21(9):3157. doi: 10.3390/ijms21093157 (PMC7247577; doi:10.3390/ijms21093157)
Supplement: Supplementary file 1 [file ijms-21-03157-s001.pdf]

International Journal of *Molecular Sciences*

MDPI

Supplementary Materials

Repurposing Tyrosine Kinase Inhibitors to Overcome Multidrug Resistance in Cancer: A Focus on Transporters and Lysosomal Sequestration

Maria Krchniakova, Jan Skoda, Jakub Neradil, Petr Chlapek and Renata Veselska

Table S1. FDA-approved TKIs and their molecular targets. (Data accessed from accessdata.fda.com).

TKI	Primary TK targets	Other targets
**afatinib** (Gilotrif)	EGFR, HER2, HER4	
**alectinib** (Alecensa)	ALK, RET	
**axitinib** (Inlyta)	VEGFR1-3	
**bosutinib** (Bosulif)	BCR-ABL, SRC, LYN, HCK	
**brigatinib** (Alunbrig)	ALK	
**cabozantinib** (Cabometyx)	HGFR, VEGFR1-3, AXL, RET, ROS1, TYRO3, MER, c-KIT, TRKB, FLT3, TIE-2	
**ceritinib** (Zykadia)	ALK, IGF-1R, InsR, ROS1	
**crizotinib** (Xalkori)	ALK, HGFR, ROS1, RON	
**dasatinib** (Sprycel)	BRC-ABL, SRC, LCK, YES, FYN, c-KIT, EPHA2, PDGFRβ	
**erlotinib** (Tarceva)	EGFR	
**gefitinib** (Iressa)	EGFR	
**ibrutinib** (Imbruvica)	BTK	
**imatinib** (Gleevec)	BCR-ABL, PDGFRα/β, c-KIT	
**lapatinib** (Tykerb)	EGFR, HER2	
**neratinib** (Nerlynx)	EGFR, HER2, HER4	
**nilotinib** (Tasigna)	BCR-ABL, PDGFRβ, c-KIT, CSF-1R, DDR	
**nintedanib** (Ofev)	PDGFRα/β, FGFR1-3, VEGFR1-3, CSF-1R, FLT3, SRC, LCK, LYN	
**osimertinib** (Tagrisso)	EGFR, HER2-4, ACK1, BLK	
**pazopanib** (Votrient)	VEGFR1-3, PDGFRα/β, FGFR1-3, c-KIT, LCK, CSF-1R, ITK	
**ponatinib** (Iclusig)	BCR-ABL	
**regorafenib** (Stivarga)	VEGFR1-3, c-KIT, PDGFRα/β, FGFR1-2, TIE-2, DDR2, TRK2A, EPHA2, FRK	RAF-1, BRAF, $BRAF^{V600E}$, SAPK2
**sorafenib** (Nexavar)	KIT, FLT3, RET, VEGFR1-3, PDGFR β	CRAF, BRAF
**sunitinib** (Sutent)	PDGFRα/β, VEGFR1-3, KIT, FLT3, CSF-1R, RET	
**vandetanib** (Caprelsa)	EGFR, VEGFR, RET, BRK, TIE-2	

ABL, Abelson murine leukemia viral oncogene homolog 1; ACK1, activated CDC42 kinase 1; ALK, anaplastic lymphoma kinase; BCR, breakpoint cluster region protein; BTK, Bruton's tyrosine kinase; BLK, B lymphoid kinase; BRK, breast tumor kinase; CSF-1R, colony stimulating factor receptor Type 1; DDR, discoidin domain receptor tyrosine kinase; EGFR, epidermal growth factor receptor; FGFR, fibroblast growth factor receptor; FLT3, Fms-like tyrosine kinase-3; FRK, Fyn-related kinase; HER, human epidermal growth factor receptor; HGFR, hepatocyte growth factor receptor; IGFR-1R, insulin-like growth foctor 1 receptor; InsR, insulin receptor; ITK, interleukin-2-inducible T cell kinase; KIT, stem cell factor receptor; LCK, leukocyte-specific protein tyrosine kinase; PDGFR, platelet-derived growth factor receptor; RET, glial cell-line derived neurotrophic factor receptor; RON, Recepteur d'Origine Nantais; SAPK, serine/threonine-protein kinase; TRK, tropomyosin receptor kinase; VEGFR, vascular endothelial growth factor receptor.

Table S2. An example of TKIs under investigation and their molecular targets. (Data accessed from pubchem.ncbi.nlm.nih.gov).

TKI	Primary TK targets	Other targets
**apatinib** (Rivoceranib)	VEGFR2, c-KIT, SRC	
**canertinib** (CI-1033)	EGFR, HER2, HER4	
**cediranib** (Recentin)	VEGFR	
**icotinib** (Conmana)	EGFR	
**linsitinib** (OSI-906)	IGF-1R	
**masitinib** (Masivet)	c-KIT, PDGFR, LCK, FAK, FGFR3	
**motesanib** (AMG 706)	VEGFR, PDGFR, c-KIT	
**quizartinib** (AC220)	FLT3	
**saracatinib** (AZD-0530)	BCR-ABL, SRC	
**tandutinib** (MLN518)	FLT3, c-KIT, PDGFR	
**telatanib** (Bay-579352)	VEGFR2-3, c-FIT, PDGFRα	
**vatalanib** (PTK787/ZK-222584)	VEGFR1-3	

ABL, Abelson murine leukemia viral oncogene homolog 1; BCR, breakpoint cluster region protein; EGFR, epidermal growth factor receptor; FAK, focal adhesion kinase; FGFR, fibroblast growth factor receptor; FLT3, Fms-like tyrosine kinase-3; HER, human epidermal growth factor receptor; IGFR-1R, insulin-like growth foctor 1 receptor; KIT, stem cell factor receptor; LCK, leukocyte-specific protein tyrosine kinase; PDGFR, platelet-derived growth factor receptor; VEGFR, vascular endothelial growth factor receptor.
